# A study on sex estimation by using machine learning algorithms with parameters obtained from computerized tomography images of the cranium

**DOI:** 10.1038/s41598-022-07415-w

**Published:** 2022-03-11

**Authors:** Seyma Toy, Yusuf Secgin, Zulal Oner, Muhammed Kamil Turan, Serkan Oner, Deniz Senol

**Affiliations:** 1 Department of Anatomy, Faculty of Medicine, Karabük University, Karabük, Turkey; 2 Department of Anatomy, Faculty of Medicine, İzmir Bakırçay University, İzmir, Turkey; 3 Department of Medical Biology, Faculty of Medicine, Karabük University, Karabük, Turkey; 4 Department of Radiology, Faculty of Medicine, İzmir Bakırçay University, İzmir, Turkey; 5 Department of Anatomy, Faculty of Medicine, Düzce University, Karabük, Turkey

**Keywords:** Anatomy, Medical research

## Abstract

The aim of this study is to test whether sex prediction can be made by using machine learning algorithms (ML) with parameters taken from computerized tomography (CT) images of cranium and mandible skeleton which are known to be dimorphic. CT images of the cranium skeletons of 150 men and 150 women were included in the study. 25 parameters determined were tested with different ML algorithms. Accuracy (Acc), Specificity (Spe), Sensitivity (Sen), F1 score (F1), Matthews correlation coefficient (Mcc) values were included as performance criteria and Minitab 17 package program was used in descriptive statistical analyses. p ≤ 0.05 value was considered as statistically significant. In ML algorithms, the highest prediction was found with 0.90 Acc, 0.80 Mcc, 0.90 Spe, 0.90 Sen, 0.90 F1 values as a result of LR algorithms. As a result of confusion matrix, it was found that 27 of 30 males and 27 of 30 females were predicted correctly. Acc ratios of other MLs were found to be between 0.81 and 0.88. It has been concluded that the LR algorithm to be applied to the parameters obtained from CT images of the cranium skeleton will predict sex with high accuracy.

## Introduction

The main purpose of forensic anthropology is to reconstruct the biological profile of deceased individuals; that is, to predict sex, age of death, lineage and height based on the remains of skeletons^1^. Forensic sex prediction has taken a large place in literature since the late 1960s and identification of sex from human skeleton has been described as an important factor, even a key element in both forensic medicine and bio-archaeological context^2–4^. Sex prediction is an indispensable part of biological profile. Anthropologist uses the biomarkers of the skeletal system that vary between sexes to determine sex^5,6^.

It is noteworthy that studies have been conducted in literature for the estimation of sex almost with all bones of the human skeleton and that the accuracy of gender determination has been researched frequently by comparing with different populations. It can be seen that various bones such as femur^2,3^, patella^7,8^, mandible^9^, calcaneus^10^, metatarsal bone and phalanx^11,12^, occipital condyle^13^, hand bones^14,15^ and sternum^16^ are used in sex prediction. It has been reported in a large number of studies in literature that cranium and pelvis bones, which are considered to be the most dimorphic areas according to skeletal parts, can be used in sex prediction by using different assessment methods^4,10,16–19^.

Identification of sex includes some inherent limitations that are affected by different factors such as ethnicity, socio-economic status, diet and geographic location. The inability to generalize the results obtained from a specific population, especially in skeletal parts such as cranium, to other populations and the need for population-specific studies increase the interest in cranium and mandible in sex determination^4,20^. For these reasons, all techniques reported for identifying sex are specific to related studies and they may not be applicable to different samples or data sets^3^.

It can be seen that methods such as discriminant analysis, machine learning algorithms (ML), support vector machine and artificial neural network are commonly used in sex prediction in which these bones are examined^2,3,7^.

ML is a modern classifier that is used extensively in the field of engineering, and it is now gradually integrated in the field of health. These algorithms are classified as supervised, unsupervised and reinforcement. Supervised learning is algorithms that match the relationship between input and output, unsupervised learning is algorithms that match the characteristics of the data about which there is no information and reinforcement leaning is the algorithms that match the input data with desired characteristics^20^. Decision Tree (DT) algorithm is one of the simple, powerful, fast and frequently used data mining classification algorithms that processes the inputs by dividing them continuously^8,21–23^. Logistic regression (LR) is a classification algorithm that uses the sigmoidal curve function to classify the relationship between output probability and parameters. Random Forest (RF) is an ensemble algorithm that can derive more than one decision tree within the system^24^. Extra Tree Classifier (ETC) is a superior method to RF, and this advantage is due to the random division of nodes and using all data as a training set^25^. Linear discriminant Analysis (LDA) is a classification algorithm that reveals the difference and relationship between classes^26^. Quadratic Discriminant Analysis (QDA) is a superior method to LDA and is a second-order parametric classifier^27^.

Computerized tomography (CT) is an imaging method that can show all tissues, especially bone tissue with sharp borders. In case of thin section, image orientation can be changed in three dimensions and can be taken to orthogonal plane. In this way, length and angle measurements can be calculated in a way that is less affected by orientation. With all these aspects, it provides superior results compared to studies carried out with more conventional osteometric devices^16^.

The aim of this study is to show the success of sex prediction by using ML with parameters obtained from CT images of cranium and mandible skeleton.

## Results

Of the 25 parameters determined, 20 (NVIC, NSVC, NNL, PC, NIVA, PNIC, VIC, NIC, RML, CML, GHGA, HML, COL, CMHA, HGGC, COIC, HGGMC, HGGMA) were found to be statistically significant between males and females (p ≤ 0.05). In 18 of these parameters which were found to be statistically significant, the average of the parameter used was higher in males, while the average of the parameter used was higher in females in 2 parameters (GHGA, CMHA) (Tables 1, 2).

**Table 1:** Comparison of parametric data of males and females.

Parameters	Sex	Mean ± std (cm)	p value
NNL	Male	2.46 ± 0.34	** < 0.01**
	Female	2.30 ± 0.31	
NNZA	Male	120.68 ± 11.12	0.35
	Female	119.55 ± 9.92	
HGGA	Male	120.22 ± 6.90	0.92
	Female	120.30 ± 6.78	
RML	Male	5.29 ± 0.51	** < 0.01**
	Female	4.54 ± 0.41	
CML	Male	7.34 ± 0.57	** < 0.01**
	Female	7.06 ± 0.49	
GHGA	Male	37.05 ± 4.60	** < 0.01**
	Female	38.99 ± 4.87	
HML	Male	8.24 ± 0.56	** < 0.01**
	Female	7.55 ± 0.58	
COLI	Male	106.61 ± 6.98	0.06
	Female	105.07 ± 7.23	
CMHA	Male	110.68 ± 10.41	** < 0.01**
	Female	114.82 ± 10.04	
HGGC	Male	12.56 ± 0.79	** < 0.01**
	Female	11.44 ± 0.69	
COIC	Male	8.61 ± 0.73	** < 0.01**
	Female	7.93 ± 0.67	
HGGMA	Male	31.15 ± 4.10	** < 0.01**
	Female	25.57 ± 3.73	

NNL: Nasion–nasal end point length, NNZA: Nasal end point–nasion–zygomatic angle, HGGA: Head of mandible– gonion–gnathion angle, RML: Ramus of the mandible length, CML: Corpus of the mandible length, GHGA: Gnathion–head of mandible– gonion angle, HML: Head of mandible–mental foramen length, COLI: Coronoid process–obliqua line–infradental angle, CMHA: Coronoid process–mandibular notch–head of mandible angle, HGGC: Head of mandible–gonion–gnathion curvature length, COIC: Coronoid process–obliqua line–infradental curvature length, HGGMA: Head of mandible–gonion–gnathion–mandibular notch area.

Significant values are in bold.

**Table 2:** Comparison of non-parametric data of males and females.

Parameters	Sex	Median (min–max), (cm)	p value
NVIA	Male	76.65 (69.54–86.03)	0.89
	Female	76.66 (43.77–86.43)	
NVIC	Male	32.41 (29.04–40.50)	** < 0.01**
	Female	30.84 (27.59–33.60)	
NSVC	Male	14.49 (12.20–18.14)	** < 0.00**
	Female	13.49 (11.43–38.97)	
ZA	Male	77.86 (62.76–95.62)	0.21
	Female	77.22 (43.65–92.02)	
PC	Male	4.67 (3.15–5.53)	** < 0.01**
	Female	4.14 (3.06–5.53)	
NIVA	Male	43.99 (14.18–73.93)	**0.03**
	Female	43.73 (37.23–51.16)	
PNIC	Male	39.47 (35.32–43.93)	** < 0.01**
	Female	37.13 (29.01–41.44)	
VIC	Male	17.78 (14.02–22.53)	** < 0.0**
	Female	17.28 (13.14–29.72)	
NFIA	Male	70.88 (45.18–81.06)	0.78
	Female	70.60 (60.79–80.64)	
NIC	Male	34.90 (31.12–38.12)	** < 0.01**
	Female	32.59 (23.45–37.39)	
GA	Male	125.31 (18.59–143.71)	0.18
	Female	125.58 (111.16–148.95)	
COL	Male	3.11 (2.13–7.36)	** < 0.01**
	Female	2.81 (1.94–3.85)	
HGGMC	Male	29.86 (17.62–33.83)	** < 0.01**
	Female	27.37 (23.08–32.79)	

NVIA: Nasion–vertex–inion angle, NVIC: Nasion–vertex–inion curvature length, NSVC: Nasion–superciliary arch–vertex curvature length, ZA: Zygomatic angle, PC: Piriform aperture curvature length, NIVA: Nasion–inion–vertex angle, PNIC: Piriform aperture–Nasal end point–inion curvature length, VIC: Vertex–inion curvature length, NFIA: Nasion–frontal tuber–inion angle, NIC: Nasal end point–inion curvature length, GA: Gonial angle, COL: Coronoid process–obliqua line length, HGGMC: Head of mandible–gonion–gnathion–mandibular notch curvature length.

Significant values are in bold.

**Figure 1:**
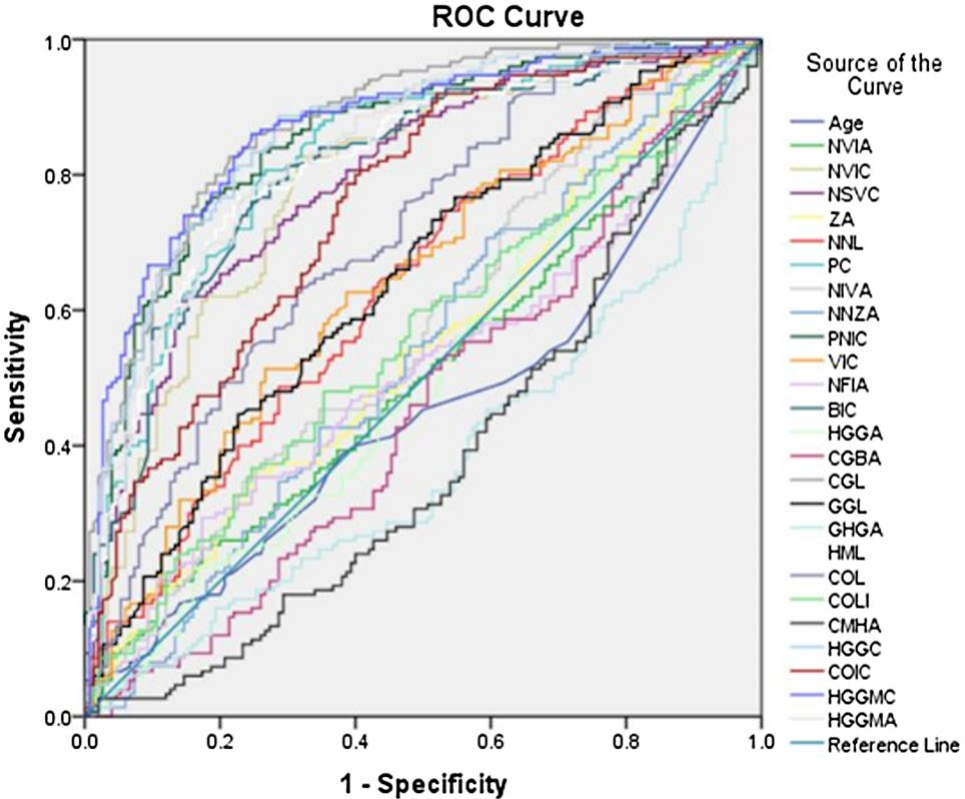
ROC curve.

**Figure 2:**
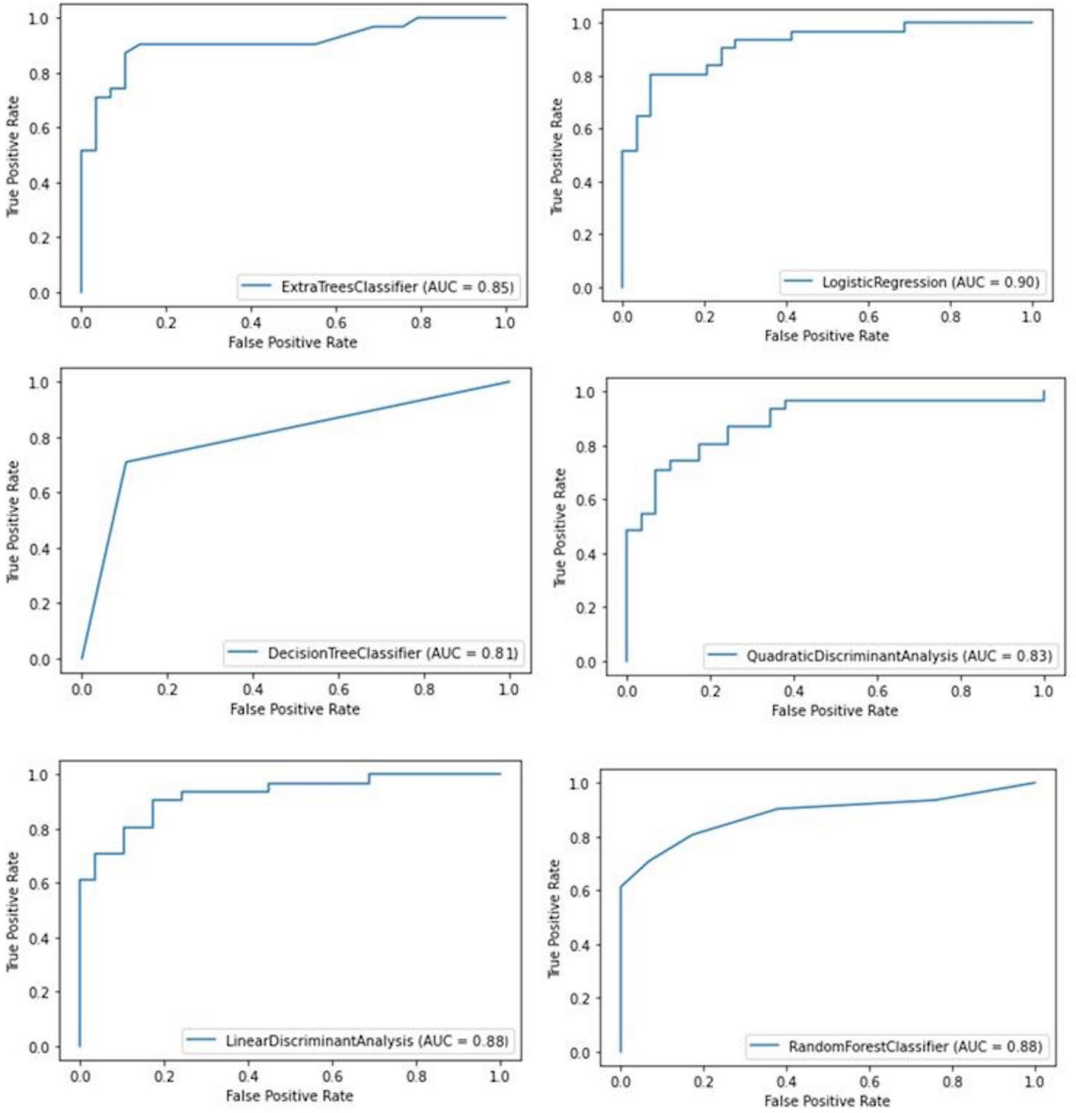
ML ROC curve.

ROC analysis was performed with the IBM SPSS (Version 21) package program to reveal the discriminative power of the parameters in distinguishing between male and female individuals, and the highest AUC ratio was obtained with the CGL parameter (Fig. 1). AUC, cut-off, p, Sen, Spe values of all parameters are given in Table 3. In addition, ROC curves and AUC values for each algorithm are given in Fig. 2.

**Table 3:** ROC result table.

Parameters	AUC (%95 CI)	Cutt-off	p	Sen	Spe
Age	0.451 (0.385–0.516)	59.5	0.141	0.467	0.54
NVIA	0.504 (0.439–0.570)	76.7	0.896	0.5	0.5
NVIC	0.791 (0.741–0.841)	31.56	0.000	0.72	0.28
NSVC	0.794 (0.744–0.845)	13.99	0.000	0.713	0.287
ZA	0.541 (0.476–606)	77.55	0.219	0.533	0.467
NNL	0.632 (0.570–0.695)	2.38	0.000	0.587	0.413
PC	0.823 (0.775–0.871)	4.36	0.000	0.753	0.247
NIVA	0.569 (0.504–0.634)	43.79	0.038	0.513	0.487
NNZA	0.536 (0.470–0.601)	120.77	0.286	0.527	0.473
PNIC	0.854 (0.810–0.897)	38.35	0.000	0.78	0.22
VIC	0.639 (0.576–0.701)	17.55	0.000	0.613	0.387
NFIA	0.509 (0.443–0.575)	70.71	0.784	0.513	0.487
BIC	0.812 (0.763–0.860)	33.59	0.000	0.753	0.247
HGGA	0.501 (0.435–0.566)	120.07	0.987	0.48	0.52
CGBA	0.456 (0.391–0.521)	125.55	0.189	0.493	0.507
CGL	0.877 (0.838–0.915)	4.86	0.000	0.8	0.2
GGL	0.642 (0.580–0.704)	7.18	0.000	0.587	0.413
GHGA	0.378 (0.315–0.442)	38.34	0.000	0.427	0.573
HML	0.815 (0.767–0.864)	7.91	0.000	0.767	0.233
COL	0.706 (0.648–0.764)	2.95	0.000	0.648	0.764
COLI	0.557 (0.492–0.622)	105.97	0.089	0.533	0.467
CMHA	0.383 (0.370–0.447)	112.88	0.000	0.42	0.58
HGGC	0.863 (0.822–0.904)	12.03	0.000	0.787	0.22
COIC	0.754 (0.700–0.808)	8.25	0.000	0.667	0.333
HGGMC	0.869 (0.827–0.910)	28.60	0.000	0.787	0.213
HGGMA	0.841 (0.797–0.884)	28.28	0.000	0.767	0.233

**Figure 3:**
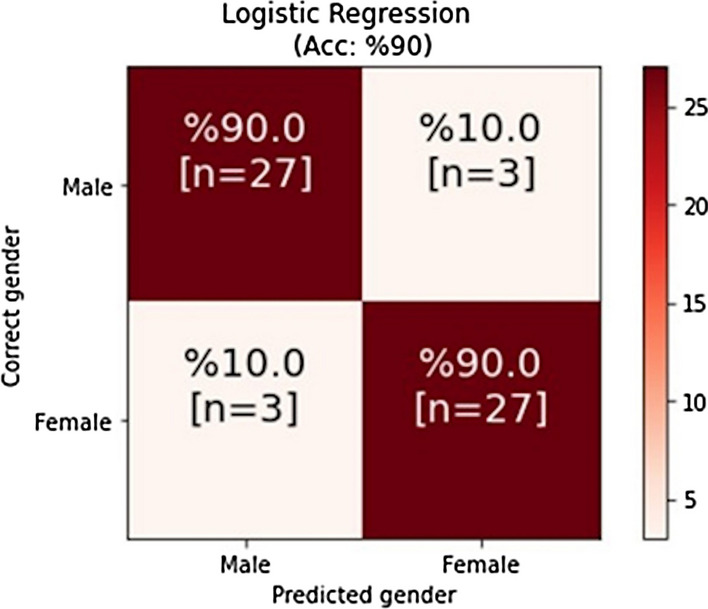
LR confusion matrix.

0.90 Acc, 0.80 Mcc, 0.90 Spe, 0.90 Sen and 0.90 F1 values were found as a result of the LR algorithm. As a result of the confusion matrix performed, 27 of 30 males and 27 of 30 females were predicted correctly (Fig. 3). Of the MLs, the highest Acc, Mcc ratio was found as 0.90, 0.80 with LR algorithm. Acc ratios of the other MLs were between 0.81 and 0.88. The coefficient of each parameter according to the LR algorithm, respectively − 5.33, 1.45, 1.05, 1.01, − 6.10, − 5.30, − 5.29, 2.84, − 4.94, 5.80, − 7.77, − 1.73, − 1.50, 1.61, − 2.28, 8.12, 1.50, 1.10, 1.22, − 2.90, 7.4, − 5.59, 4.03, 4.20, − 3.01 as was found, and HGGMA, PC, BIC HGGA, CMHA, HGGC parameters were statistically significant in terms of gender.

0.88 Acc, 0.77 Mcc, 0.88 Spe, 0.88 Sen, 0.88 F1 values were found as a result of LDA algorithm and 26 of 30 males and 27 of 30 females were predicted correctly as a result of confusion matrix. 0.83 Acc, 0.67 Mcc, 0.83 Sep, 0.83 Sen, 0.83 F1 values were found as a result of QDA algorithm and 24 of 30 males and 26 of 30 females were predicted correctly as a result of confusion matrix. 0.88 Acc, 0.77 Mcc, 0.88 Spe, 0.88 Sen, 0.88 F1 values were found as a result of RF algorithm and 24 of 30 males and 27 of 30 females were predicted correctly as a result of confusion matrix. 0.85 Acc, 0.70 Mcc, 0.85 Spe, 0.85 Sen, 0.85 F1 values were found as a result of ETC algorithm and 24 of 30 males and 27 of 30 females were predicted correctly as a result of confusion matrix. 0.81 Acc, 0.67 Mcc, 0.81 Spe, 0.81 Sen, 0.81 F1 values were found as a result of DT algorithm and 24 of 30 males and 23 of 30 females were predicted correctly as a result of confusion matrix.

In addition, in terms of the reliability of our study, the tenfold cross-validation estimation values of the algorithms are also included. As a result of tenfold cross validation, Acc ratio of 87.766 ± 0.819 with LR algorithm, Acc ratio of 87.733 ± 0.410 with LDA algorithm, Acc ratio of 86.533 ± 0.592 with QDA algorithm, Acc ratio of 85.766 ± 1.045 with RF algorithm, Acc ratio of 77.200 ± 1.970 with ETC algorithm, Acc ratio of 80,266 ± 1.396 was obtained with the DT algorithm (Table 4).

**Table 4:** Tenfold cross validation results (%Acc).

Testing set	LR	LDA	QDA	RF	ETC	DT
1	87	87.33	85.33	85.33	75.33	78.67
2	86.33	87.67	86.67	85	75.67	81
3	86	87	86.33	86.67	74	83.67
4	87.67	88.33	87.33	85.33	78	80.67
5	86.33	87.67	86.67	87.67	80.33	80.33
6	85.33	88	86	84.33	78	79.33
7	87.67	88	86.67	85.33	77.33	80
8	86.33	87.33	87.33	87	78.67	80.33
9	87.67	88	86.67	86	75.67	79.33
10	87.33	88	86.33	85	79	79.33
Mean	87.766	87.733	86.533	85.766	77.200	80.266
Std	0.819	0.410	0.592	1.045	1.970	1.396

**Figure 4:**
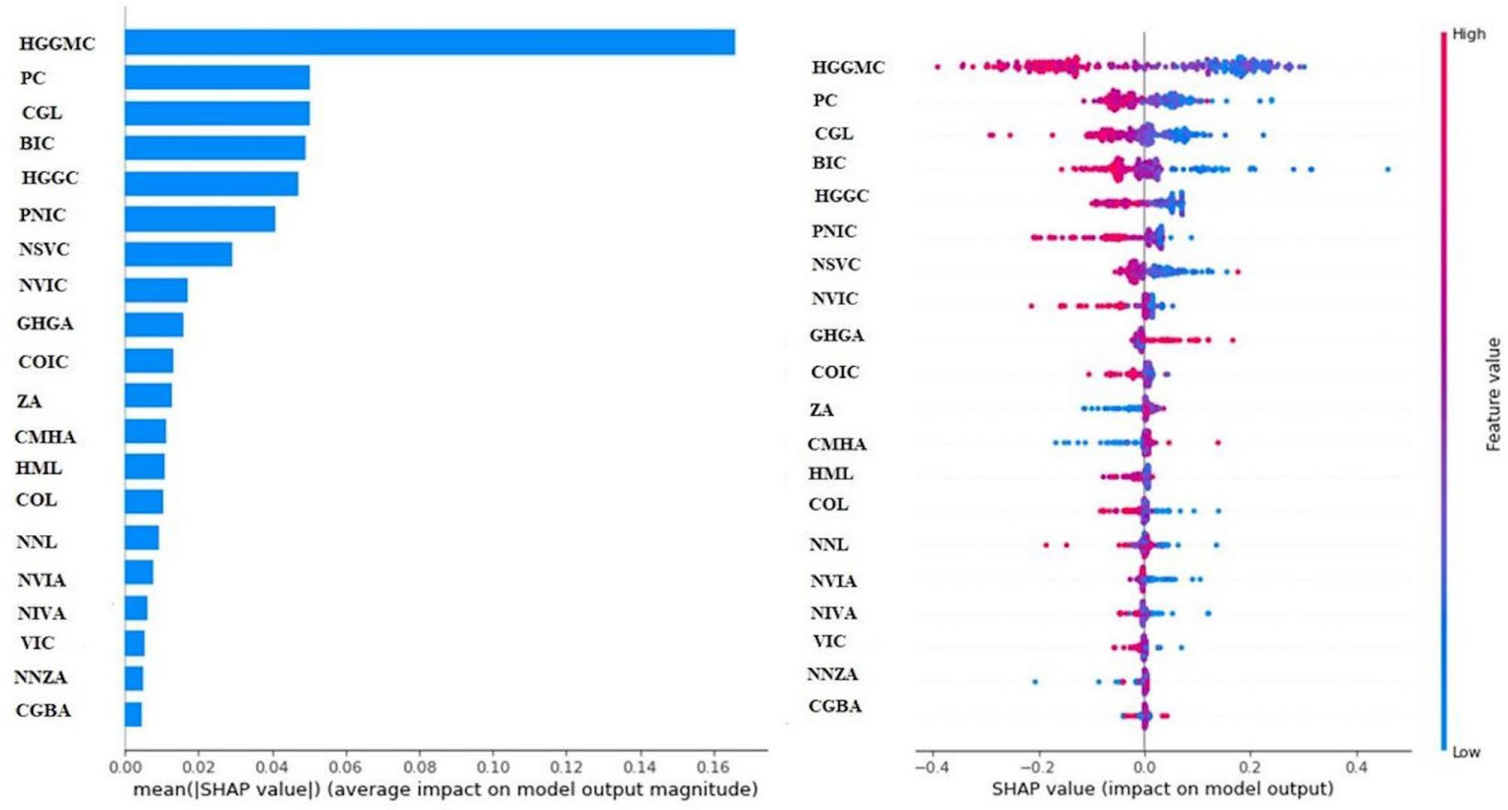
RF algorithm SHAP explanatory image.

In our study, the SHAP explanatory model of the RF algorithm was used to reveal the contribution of the parameters to the general algorithm, and it was found that the first five contributions were found to be with the parameters HGGMC, PC, GGL, HGGA, HGGC (Fig. 4).

## Discussion

The aim of this study is to test whether sex identification can be made by using ML with the parameters obtained from cranium and mandible CT images taken to orthogonal plane. In the statistical analysis performed, NVIC, NSVC, NNL, PC, NIVA, PNIC, VIC, NIC, RML, CML, HML, COL, HGGC, COIC, HGGMC, HGGMA parameters were found to be statistically significant in distinguishing between sexes (p ≤ 0.05). Of the MLs tested, 0.90 Acc, 0.80 Mcc, 0.90 Spe, 0.90 Sen, 0.90 F1 values were found as a result of LR algorithm. It was found that 27 of 30 males and 27 of 30 females were predicted correctly as a result of confusion matrix. Acc ratios of other MLs were found to be between 0.81 and 0.88. Working in small datasets, lack of external validation, and not working in different populations are the limitations of our study.

Forensic anthropologists constantly try to improve skeletal identification methods by using various methods in various parts of the skeleton or by developing new methods to determine gender^4^. Pelvis and cranium are known as the most dimorphic skeletal parts and they form the basis of sex determination researches^4,10,17–19^. Bertsatos et al.^19^ reported that they predicted sex with an Acc ratio of 0.71–0.90 in total according to the results of the discriminant function analysis they carried out with the parameters taken from the cranium. Franklin et al.^28^ and Dayal et al.^29^ reached Acc ratios of 0.88- 0.90 and 0.80- 0.85, respectively according to the results of the discriminant function analysis they carried out with the parameters taken from the cranium. In this study, 0.90 Acc, 0.80 Mcc, 0.90 Spe, 0.90 Sen, 0.90 F1 results were found as a result of LR algorithm. Since the ML results included Mcc value which can evaluate Acc, Spe, Sen values together and which shows the reliability of algorithm, it is thought that reliability and accuracy were tested with various methods and reliable results were found in the study^12^.

While discriminant function analysis is one of the most widely used methods in forensic and archaeological cases for the determination of sex in literature, it is known that error rates are always different from 0%^2^. The fact that the MLs used in the present study were trained as 80% training and 20% test set increases the prediction reliability of the study and makes it more advantageous when compared with discriminant analysis.

CT is preferred for providing advantage in the measurement of missing and damaged parts by making bone measurements very close to original and allowing for the reconstruction of each bone part, unlike conventional osteometry devices (calliper, odontometer, digital distance meter)^16,22^. As far as we know, studies that associate parameters taken from cranium and mandible on orthogonal plane with ML based sex prediction are very limited. Even if CT is used in current studies, the results can show differences because the orientation of the image is not converted to the orthogonal plane since especially angular measurements are parameters affected by orientation.

In their study they predicted sex from cranium by using CT, Gillet et al.^30^ used geometric morphometric model in their study and reported that they reached 0.90 Acc ratio for skull model. Zaafrane et al.^31^ reported that they estimated sex with an Acc ratio of 0.90 from parameters of cranium in CT images they analysed by using basic statistical methods. These differences in results can be explained with the fact that the evaluation of sexually dimorphic features depend on group specific standards and skeletal characteristics differ among different populations, as well as the methodological methods used and differences in statistical analyses.

Imaizumi et al.^32^ They used the support vector machine in their study in which they examined 100 skull skeletons and obtained a gender prediction rate of over 90% with 10% cross validation. In this study, we use image-based CNN, SVM, etc. We did not choose algorithms. The reason for this is due to the selection of only anthropometric points, not the entire cranium skeleton. Anthropometric points were measured manually using the Horos Project program and the results were used as ML algorithm input. Because image-based algorithms will produce a result by learning all the points of the given cranium skeleton.

It has been reported in literature that the possibility of removing the mandible intact is high^33^. The reason for this is the fact that the presence of a dense compact bone layer in the mandible makes it durable and therefore more likely to be found intact^34^. It is reported in literature that the measurements taken from the mandible are generally obtained from panoramic radiography images and that these images are affected by orientation^35^. According to the results of studies in which only the measurements taken from mandible are evaluated, an Acc ratio between 0.60 and 0.88 seems to be a reliable structure for sex prediction^29,35–37^. In this study, combining the parameters taken from the mandible with the cranium strengthened gender prediction. RML, CML, GCGA, CFL, PLL, PICA, CGC, PLIC, CGGIC, CGGIA parameters taken from the mandible were found to be statistically significant in sex identification.

Since the identity of individuals should be predicted quickly and accurately in events such as war, natural disasters and fire, which deeply affect the society, the CT technology and MLs used in the present study show that prediction time can be minimized and high accuracy can be obtained. Considering the high Acc ratio found as a result of LR algorithm, it is thought that the present study will strengthen and contribute to studies related with sex prediction.

## Materials and methods

### Image set and population

The study was conducted at Karabük University Training and Research Hospital, Department of Radiology after 2020/363 coded approval of Karabük University Faculty of Medicine non-interventional clinical research ethics committee.

The image set in the study consisted of the CT images of 150 male and 150 female individuals whose ages ranged between 20 and 65. Individuals with any surgical operation or pathology of the cranium skeleton were excluded from the study. Average age of the males was 54 (min 20, max 65), while average age of the females was (min 21, max 65). No statistically significant difference was found between the average ages of males and females (p = 0.395).

### Multidetector CT (MDCT) protocol

Radiological images used in the study were obtained from CT images with a section thickness of 5 mm taken in supine position by using a 16-row MDCT scanner (Aquilion 16; Toshiba Medical Systems, Otawara, Japan) in the department of radiology of a Karabük University Training and Research Hospital. Scanning protocol values were tube voltage: 120 kV, gantry rotation: 0.75 s and pitch: 1.0 mm.

### Image analysis

**Figure 5:**
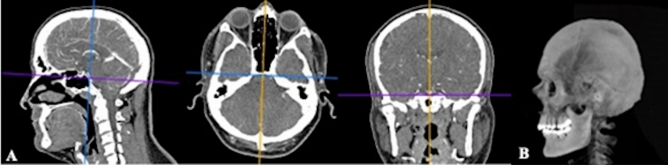
(**A**) Sagittal, transversal and coronal images brought to orthogonal plane, (**B**) Overlapped image.

The images obtained were transferred to Horos Medical Image Viewer (Version 3.0, USA) program, which is a personal workstation in Digital Imaging and Communications in Medicine (DICOM) format. Images in sagittal, transversal and coronal planes were obtained from the transferred images by using 3D Curved Multiplanar Reconstruction (MPR). The line passing through the nasion and inion points of the images in these three planes was determined and all images were brought to the orthogonal plane (Fig. 5A). Later, CT images brought to orthogonal plane were overlapped by increasing their section thicknesses (Fig. 5B).

**Figure 6:**
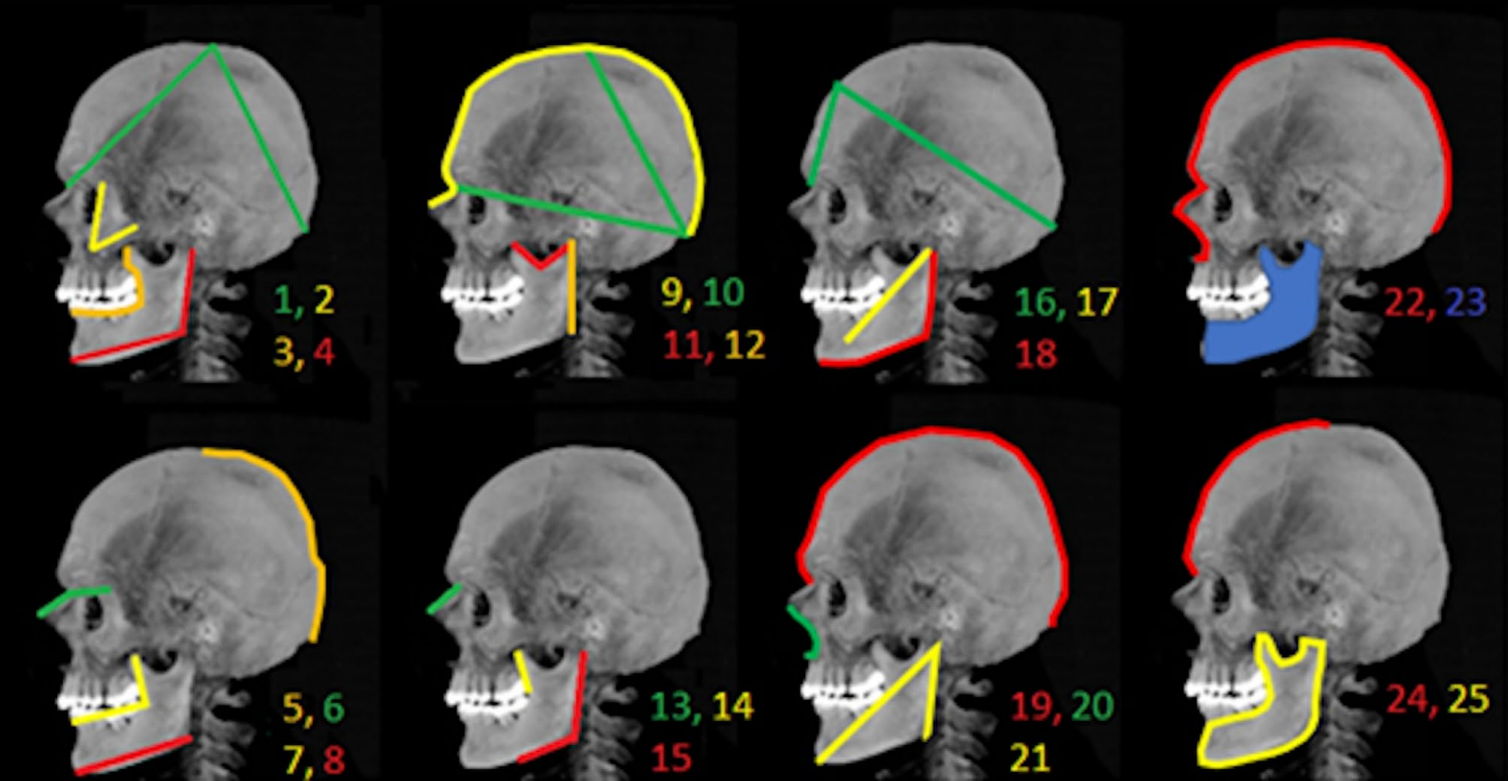
Demonstration of parameters (1: NVIA, 2: ZA, 3: COIC, 4: HGGA, 5: VIC, 6: NNZA, 7: COLI, 8: CML, 9: NIC, 10: NIVA, 11: CMHA, 12: RML, 13: NNL, 14: COL, 15: GA, 16: NFIA, 17: HML, 18: HGGC, 19: NVIC, 20: PC, 21: GCGA, 22: PNIC, 23: HGGMA, 24: NSVC, 25: HGGMC).

Length, angle, area and curvature length measurements of the anatomic points of the overlapped images were performed. These parameters and their abbreviations are listed below in Tables 5, 6 and 7. Demonstration of all evaluated parameters is shown in Fig. 6.

**Table 5:** Length parameters and abbreviations.

Parameters	Abbreviations
Ramus of the mandible length	RML
Corpus of the mandible length	CML
Head of mandible–mental foramen length	HML
Coronoid process–obliqua line length	COL
Nasion–nasal end point length	NNL

**Table 6:** Angle parameters and abbreviations.

Parameters	Abbreviations
Head of mandible–gonion–gnathion angle	HGGA
Gonial angle	GA
Gnathion–head of mandible–gonion angle	GHGA
Coronoid process–obliqua line–infradental angle	COLI
Coronoid process–mandibular notch**–**head of mandible angle	CMHA
Nasion–vertex–inion angle	NVIA
Zygomatic angle	ZA
Nasion–inion–vertex angle	NIVA
Nasal end point–nasion–zygomatic angle	NNZA
Nasion–frontal tuber–inion angle	NFIA

**Table 7:** Curve lenght-area parameters and abbreviations.

Parameters	Abbreviations
Head of mandible–gonion–gnathion curvature length	HGGC
Coronoid process–obliqua line–infradental curvature length	COIC
Head of mandible–gonion–gnathion–mandibular notch curvature length	HGGMC
Nasion–vertex–inion curvature length	NVIC
Nasion–superciliary arch–vertex curvature length	NSVC
Piriform aperture curvature length	PC
Piriform aperture–nasal end point–inion curvature length	PNIC
Vertex–inion curvature length	VIC
Nasal end point–inion curvature length	BIC
Head of mandible–gonion–gnathion–mandibular notch area	HGGMA

### Machine learning algorithms

In this study, scikit-learn model (Version 0.20.0) in Python programming language (Version 3.7.1) was used to make ML modelling^38^. ML modelling was performed by using i7, 8 GbHp-Folio 1040 model computer. Decision Tree (DT), Random Forest (RF), Logistic Regression (LR), Linear Discriminant Analysis (LDA), Quadratic Discriminant Analysis (QDA), Extra Tree Classifier (ETC) algorithms were used. The dataset was mixed by shuffling, and the first 80% (240 measurements) was designated as the training set, while the last 20% (60 measurements) was designated as the test set. In addition, tenfold cross validation accuracy values are also included in terms of the reliability of our study.

### Performance criteria

Accuracy (Acc), Specificity (Spe), Sensitivity (Sen), F1 score (F1), and Matthews correlation coefficient (Mcc) values were included as performance criteria.

1 $$\begin{aligned} \mathrm{Acc} & =\frac{\mathrm{TP}}{\mathrm{TP}+\mathrm{FN}+\mathrm{FP}+\mathrm{TN}}\\ \mathrm{Sen} & =\frac{\mathrm{TP}}{\mathrm{TP}+\mathrm{FN}} \\ Spe & =\frac{TN}{TN+FP}\\ \mathrm{Mcc} & =\frac{\mathrm{TP}\times \mathrm{TN}-\mathrm{FP}\times \mathrm{FN}}{\sqrt{(\mathrm{TP}+\mathrm{FP})\times (\mathrm{TP}+\mathrm{FN})\times (\mathrm{TN}+\mathrm{FP})\times (\mathrm{TN}+\mathrm{FN})}}\\ \mathrm{F}1 & =2\frac{\mathrm{Specificity}\times \mathrm{Sensitivity}}{\mathrm{Specificity}+\mathrm{Sensitivity}} \end{aligned}$$

TP: True positive, TN: True negative, FP: False positive, FN; False negative.

### Statistical analysis

Mean, standard deviation, minimum and maximum values were included in the descriptive statistics of each data according to gender groups. Normality test Anderson Darling test was applied to each parameter and it was checked whether the data were normally distributed. Two simple T test was applied to parametric data and Mann–Whitney U test was applied to nonparametric data and p ≤ 0.05 value was considered as statistically significant. In order to reveal the differences of the parameters in terms of gender, ROC analysis was performed and the ROC curve was included. Minitab 17 and IBM SPSS (Version 21) package program was used in analyses.

### Ethical considerations

This retrospective study was initiated with the 2020/363 decision of the Karabük University Faculty of Medicine non-interventional clinical research ethics committee.

### Ethical approval

The present study was approved by Karabük University Faculty of Medicine Local Non-Interventional Clinical Trials Ethics Committee with the protocol number 2020/363. All procedures performed in studies involving human participants were in accordance with the ethical standards of the institutional and/or national research committee and with the 1964 Helsinki declaration and its later amendments or comparable ethical standards.

### Informed consent

This study is retrospective and based on images taken from the hospital archive system. Therefore, the requirement for informed consent for the study was waived by the Karabük University Faculty of Medicine Local Non-Interventional Clinical Trials Ethics Committee.

### Conference presentation

This study was presented as an oral presentation at the 21st National Anatomy Congress in Turkey.
